# Central administration of insulin-like growth factor-I decreases depressive-like behavior and brain cytokine expression in mice

**DOI:** 10.1186/1742-2094-8-12

**Published:** 2011-02-09

**Authors:** Sook-Eun Park, Robert Dantzer, Keith W Kelley, Robert H McCusker

**Affiliations:** 1 Integrated Immunology and Behavior Program University of Illinois at Urbana-Champaign Urbana, Illinois 61801-3873, USA; 2 Neuroscience Program University of Illinois at Urbana-Champaign Urbana, Illinois 61801-3873, USA; 3 Department of Animal Sciences University of Illinois at Urbana-Champaign Urbana, Illinois 61801-3873, USA; 4 Department of Pathology University of Illinois at Urbana-Champaign Urbana, Illinois 61801-3873, USA

## Abstract

Exogenous administration of insulin-like growth factor (IGF)-I has anti-depressant properties in rodent models of depression. However, nothing is known about the anti-depressant properties of IGF-I during inflammation, nor have mechanisms by which IGF-I alters behavior following activation of the innate immune system been clarified. We hypothesized that central IGF-I would diminish depressive-like behavior on a background of an inflammatory response and that it would do so by inducing expression of the brain-derived neurotrophic factor (BDNF) while decreasing pro-inflammatory cytokine expression in the brain. IGF-I (1,000 ng) was administered intracerebroventricularly (i.c.v.) to CD-1 mice. Mice were subsequently given lipopolysaccharide i.c.v. (LPS, 10 ng). Sickness and depressive-like behaviors were assessed followed by analysis of brain steady state mRNA expression. Central LPS elicited typical transient signs of sickness of mice, including body weight loss, reduced feed intake and decreased social exploration toward a novel juvenile. Similarly, LPS increased time of immobility in the tail suspension test (TST). Pretreatment with IGF-I or antidepressants significantly decreased duration of immobility in the TST in both the absence and presence of LPS. To elucidate the mechanisms underlying the anti-depressant action of IGF-I, we quantified steady-state mRNA expression of inflammatory mediators in whole brain using real-time RT-PCR. LPS increased, whereas IGF-I decreased, expression of inflammatory markers interleukin-1ß (IL-1ß), tumor necrosis factor-(TNF)α, inducible nitric oxide synthase (iNOS) and glial fibrillary acidic protein (GFAP). Moreover, IGF-I increased expression of BDNF. These results indicate that IGF-I down regulates glial activation and induces expression of an endogenous growth factor that shares anti-depressant activity. These actions of IGF-I parallel its ability to diminish depressive-like behavior.

## Background

Recent studies have unequivocally linked activation of the innate immune system with development of metabolic, subjective and behavioral components of sickness. Peripheral or central administration of the cytokine inducer LPS induces transient anorexia, social isolation, general malaise, an increase in non-rapid eye movement sleep and fever [1]. All of these symptoms are dependent on neuroinflammation and the production of pro-inflammatory cytokines within the brain. Sustained activation of the innate immune system can lead to development of depressive disorders [2]. Several conditions, such as aging and obesity, and diseases, such as rheumatoid arthritis, atherosclerosis and congestive heart failure, are associated with an increased prevalence of depressive disorders. These conditions have a common underlying chronic inflammatory component [3]. Indeed, elevated levels of circulating pro-inflammatory cytokines, including TNFα, IL-6 and IL-1β, are frequently observed in patients with depression [4]. Although an associative link between neuroinflammation and sickness behavior is now widely accepted, the above studies do not provide a cause-effect relationship between neuroinflammation and development of depression disorders.

There is increasing evidence that development of depression can be associated with activation of the innate immune system [5,6]. In particular, cytokine therapy for certain types of cancer and viral infections induces development of depressive symptoms in a significant percentage of the population under consideration [7,8]. Humans exposed to low-dose endotoxin elicited a depressed mood that correlated with cytokine secretion. Interestingly this low dose of endotoxin did not elicit symptoms of sickness [9]. A similar reduction in mood occurs in humans injected with a typhoid vaccine, and this decline significantly correlates with an increase in IL-6 secretion and enhanced activity within subgenual anterior cingulate cortex [10]. These findings provide a direct cause-effect relationship between neuroinflammation and depression and a distinction between overt sickness and depression. At the preclinical level, acute and chronic activation of the immune system reliably induces depressive-like behavior of mice. LPS induces transient sickness followed by depressive-like behavior, increased immobility in the forced swim test (FST) and the TST. These behaviors are reversed by anti-depressants and by minocycline which attenuates neuroinflammation [11,12]. These and other studies clearly suggest that development of anti-inflammatory regimes would be a viable strategy as a potential therapeutic for inflammation-associated depressive disorders.

IGF-I, a neurotrophic hormone, elicits a broad spectrum of biological activities [13]. However, few studies have been reported that describe an anti-inflammatory action of IGF-I. IGF-I decreased expression of pro-inflammatory cytokines following treatment with galactosamine plus LPS, which results in IGF-I mediated liver protection [14] and reduced atherosclerosis progression in ApoE mice [15]. Also, IGF-I gene transfer attenuated glial activation and tau hyper-phosphorylation following spinal cord injury [16]. These studies and others illustrate that IGF-I may be anti-inflammatory. However, the positive effects in these studies may reflect the ability of IGF-I to modulate macrophage or neutrophil infiltration into tissues, rather than a direct anti-inflammatory action on the immune system, which would be evident as decreased cytokine expression per cell. Only a couple of *in vitro *studies have shown a direct anti-inflammatory potential for the IGF system. Microglia-derived IGF-II, which acts similar to IGF-I, inhibited TNFα-activation of JNK in oligodendrocytes [17] and IGF-I increased dephosphorylation of IкB in astrocytes; thereby diminishing NF-кB activity following TNFα exposure [18,19]. IGF-I also depressed TNFα-induced NF-кB activation in colonic adenocarcinoma cells [6,20]. Importantly, IGF-I mediates the anti-inflammatory actions of the neural cell adhesion molecule (NCAM) mimetic, FGL, thus tempering glia activation associated with aging and glial activation following treatment with interferon-(IFN)γ [21]. Similarly, IGF-I decreased IFNγ-induced and age-associated microglial activation, IL-1β induction and deficit in long-term potentiation [22]. Exogenous IGF-I, given i.c.v., can even temper the development of sickness behavior induced by either TNFα or LPS [23-25]. Taken together, these data indicate that IGF-I can attenuate an inflammatory response both at the cellular and subcellular levels and antagonize neuroinflammatory-induced behavioral changes.

To date, IGF-I has been tested for anti-depressant activity using naïve rodents and following chronic mild unpredictable stress (CUS). Chronic IGF-I administration, s.c. at 50 μg/kg/d, decreased immobility in the FST of naïve mice, decreased consumption latencies in the novelty-induced hypophagia test of naïve mice and increased sucrose consumption by mice following CUS [26-29]; all suggesting anti-depressant activity. In the same study, exercise-induced improvement in the FST was blocked by an anti-IGF-I antibody [29], suggesting that exercise has an anti-depressant activity that is dependent on IGF-I. Again with naïve mice, IGF-I and NBI-31772 (a drug which frees endogenous IGF-I from a natural inhibitor) decreased immobility in the TST. The action of IGF-I and NBI-31772 was blocked by the IGF type 1 receptor antagonist JB1 [28]. These actions were independent of changes in general locomotor activity, which is used an index of sickness [30,31], suggesting behavioral specificity. Using naïve rats, a single i.c.v. injection of 1 μg IGF-I decreased immobility in the FST [26]. JB1 blocked the decreased immobility in the FST that was present after a single i.c.v. 1 μg dose of IGF-I [27]. These data clearly indicate that IGF-I has anti-depressant activity using a variety of measures (ex., FST, TST, sucrose consumption; which parallel each other as reliable indices of depressive-like behaviors of rodents treated with IGF-I).

However, IGF-I has not yet been evaluated for anti-depressive actions on the important background of acute neuroinflammation. Here we have filled this void by defining the ability of exogenous IGF-I to modulate depressive-like behavior of LPS-challenged mice. LPS and IGF-I were both administered i.c.v. to directly test if IGF-I was able to modulate a central inflammatory response, independent of actions at the periphery. Based on the association of neuroinflammation with depression, we then examined whether the anti-depressive effect of IGF-I was associated with its ability to temper the neuroinflammatory processes within the brain. Our data show that central IGF-I significantly impairs development of depressive-like behavior and this action was related to an anti-inflammatory response in the brain measured as measured by a reduction in expression of inflammatory markers. Moreover, IGF-I induced expression of BDNF, which has well-characterized anti-depressant activity. These results provide strong evidence that IGF-I within the brain tempers depressive-like behavior in both naïve and LPS-challenged mice.

## Methods

### Animals

Male CD-1 mice, 7 to 8 weeks old, were purchased from Charles River Laboratory International, Inc. (Wilmington, MA). Upon arrival, mice were allowed to acclimate to the new environment for 2 weeks. Mice were group housed in ventilated cages and maintained in the standard colony room under a 12:12 h reversed light: dark cycle (lights off at 10:00 h). Juvenile 3 to 4 wk of age C57BL/6J mice from our in-house breeding colony were used for tests of social exploration. All mice were provided feed and water *ad libitum*. Animal care and procedures were conducted with the approval of the University of Illinois' Institutional Animal Care and Use Committee.

### Surgery

Surgery for cannula placement was performed under aseptic conditions. Each CD-1 mouse was anesthetized with a mixture of ketamine (100 mg/kg body weight) and xylazine (10 mg/kg body weight); post-surgical pain was attenuated using buprenorphine (0.05 mg/kg body weight). The head of each mouse was secured in a stereotaxic instrument (David Kopf Instruments, Tujunga, CA). A 26-gauge stainless-steel guide cannula (Plastics One Inc, Roanoke, VA) was unilaterally implanted above the lateral cerebral ventricle of the brain 0.6 mm posterior and 1.3 mm lateral to the bregma. The pedestal extended 1.3 mm below the skull at the point of entry. Each implanted guide cannula was secured with "cold cure" Teets denture mixture (Co-oral-lte Dental MFG Co, Diamond springs, CA). After surgery, mice were individually housed in conventional cages and allowed a 2-week recovery period before treatment.

### Treatments

Mice were handled for at least 5 days prior to treatment to minimize restraint stress during the injections. Mice were transferred to the behavioral test room 2 or 3 days before treatment. Experimental mice were 11 to 12 weeks of age on the day of treatment, which were all administered at the end of light phase. Recombinant human IGF-I (GroPep, Adelaide, Australia) was prepared at 1,000 ng/μl and LPS (serotype 0127:B8, Sigma, St. Louis, MO) was prepared at 10 ng/μl. Preliminary dose response experiments revealed that these were the optimal doses of LPS and IGF-I for reliably inducing depressive-like behavior and decreasing depressive-like behavior, respectively. The 1 μg dose of IGF-I was the same as the i.c.v. dose that had anti-depressant activity [26,27] and that we had previously shown to have positive behavioral effects against central LPS [23]. LPS and IGF-I were administered into the lateral cerebral ventricle with a 33-gauge stainless-steel guide internal cannula with a 1 μl total volume. IGF-I or PBS was administered 30 min prior to LPS or PBS for the 4 treatment combinations: PBS/PBS (Control), IGF-I/PBS (IGF-I), PBS/LPS (LPS) and IGF-I/LPS (IGF-I + LPS). Fluoxetine and desipramine (Sigma) were prepared at 4 and 2 mg/ml and administered i.p. at 100 μl/10 gm body weight for a final dose of 40 and 20 mg/kg body weight, respectively. PBS served as the excipient control. Fluoxetine and desipramine were given as a single injection 30 min prior to the TST.

### Sickness response

Sickness was assessed by measuring changes in body weight, feed intake and social exploration. Body weight and feed weight were recorded the day before treatment, immediately before treatment and prior to each behavioral assessment. Feed intake was estimated as the disappearance of feed. For social exploration, a C57BL/6J juvenile was confined to a 8 × 8 × 11.5 cm wire cage and placed in the corner of the experimental mouse's home cage. The home cage lid was replaced by a Plexiglas plate for ease of observation. The time spent by the experimental mouse showing exploratory behavior towards the caged juvenile was recorded by a trained person blind to treatment. Each experimental mouse was recorded for 5 min. All behavioral assessments were performed during the dark phase of the light cycle under red light illumination. A white noise machine (Marpac soundscreen) was used to minimize interference from external sounds. Baseline social activity was assessed 2 h into the dark cycle the day before treatment (i.e. equivalent time of day to the 2 h experimental time point).

### Depressive-like behavior

Depressive-like behavior was measured as duration of immobility in the TST. The TST was performed as previously described [12] using the Mouse Tail Suspension Package (MED-TSS-MS; Med Associates, St Albans, VT). In brief, adhesive tape was attached to the tail of the experimental mouse for suspension from a hook connected to a strain gauge. Generated force from the mouse's struggle was recorded in real time during a 10 min test session. Program settings were start trigger = 10, gain = 4, lower threshold = 3 and upper threshold = 100. Mice were considered immobile if the recorded force was below the lower threshold. In order to minimize the number of behavioral manipulations, we specifically chose to use the TST as a measure of depressive-like behavior. We [12] and others [32] have previously reported nearly identical results using either the TST or forced swim test.

### Tissue preparation, mRNA extraction and real-time RT-PCR

Mice were euthanized in a carbon dioxide filled chamber and transcardially perfused with 30 ml of cold PBS. The brain was excised and frozen on dry ice. The brains were stored at -80°C until pulverized with pestle and mortar that were pre-chilled with dry ice. Pulverized tissue was homogenized using an ultra sonicator and TRIzol reagent (Invitrogen Life Technologies, Carlsbad, CA). Chloroform was added to the dissolved tissue followed by acid phenol for phase separation. The aqueous phase was transferred to isopropanol to precipitate RNA. The RNA was washed with 75% ethanol and re-suspended with diethyl polycarbonate-treated water. RNA concentration was estimated using a Nanodrop ND-1000 spectrophotometer (Nanodrop Technologies, Inc. Wilmington, DE). To synthesize cDNA, RNA was reverse transcribed using High Capacity cDNA Reverse Transcription Kits according to the manufacturer's instructions (catalog no. 4368813, Applied Biosystems, Foster city, CA). Real-time reverse transcription PCR was performed to quantify the steady-state mRNA level of targeted genes as described previously [12]. In brief, primers were purchased from Applied Biosystems (Foster City, CA), and amplification was performed with a Prism 7900 (Applied Biosystems) using TaqMan universal PCR master mix (Applied Biosystems, catalog no. 4305719) and 2 μg of sample cDNA. The endogenous housekeeping gene, GAPDH, was used to normalize target gene expression. Relative and quantitative changes of amplified target cDNAs were analyzed by comparing 2^-ΔΔCts^, where Ct is the cycle threshold. A brief description of the target genes and primers are listed in Table 1.

**Table 1:** PCR targets

Protein	**Gene**_**(transcript)**_	Primary Expressing Cells	Function	Ct	Catalog #
TNFα	Tnf	Microglia	Pro-Inflammatory Cytokine	30	Mm00443258_m1
IL-1ß	Il1b	Microglia	Pro-Inflammatory Cytokine	**28**	Mm00434228_m1
iNOS	Nos2	Microglia	Nitric Oxide Generation	30	Mm00440485_m1
IL-10	Il10	Microglia, Astrocytes	Anti-Inflammatory Cytokine	35	Mm00439616_m1
IL-4	Il4	Microglia	Anti-Inflammatory Cytokine	38	Mm00445260_m1
IL-6	Il6	Astrocytes, Microglia	Pro-Inflammatory Cytokine	32	Mm00446190_m1
GFAP	Gfap	Astrocytes	Cytoskeletal	**18**	Mm00546086_m1
BDNF	Bdnf _(I-IX)_	Neuronal Soma	Growth Factor	**26**	Mm01334047_m1
BDNF	Bdnf _(VI-IX)_	Neuronal Dendrites-Soma > Astrocytes	Growth Factor	**25**	Mm01334042_m1
IGF-I	Igf1 _(Ea)_	Neurons, Activated Glia	Growth Factor	**27**	Mm00710307_m1
IGF-I	Igf1 _(Eb)_	Neurons, Activated Glia	Growth Factor	30	Mm00439561_m1
COX2	Ptgs2	Endothelial cells	Prostanoid Synthesis	**25**	Mm00478372_m1
GAPDH	Gapdh	All Cells	Glycolysis	**17**	Mm99999915_g1

Gene expression pattern was designed to initiate work to define the mechanism by which IGF-I affects behavior. To do this; steady state expression of a panel of genes were quantified. Although not directly translatable to mRNA levels, average Ct values for control mouse samples are provided for each transcript to give an indication of their relative abundances. Gene expression with Ct values of ≥ 35 were considered as low expression genes (right justified), genes with Ct values between 30 and 34 were of medium expression (center justified, underlined) and those with Ct values of ≤ 29 were highly expressed (left justified, bold-faced).

### Statistical analysis

All data were expressed as mean ± SEM; n = 15-16 mice per mean for behavior and n = 11-12 mice per mean for real time rtPCR except the fluoxetine/desipramine experiment with an n = 8. Data were analyzed by a two-way (LPS × IGF-I; LPS × anti-depressants) analysis of variance using StatView (SAS Institute Inc., San Francisco, CA) except the data for social investigation, body weight and feed disappearance which were analyzed by repeated-measures ANOVA. Tukey's HSD was used for *post hoc *analysis if an interaction was significant.

## Results

### IGF-I decreases duration of immobility in the presence and absence of LPS

**Figure 1:**
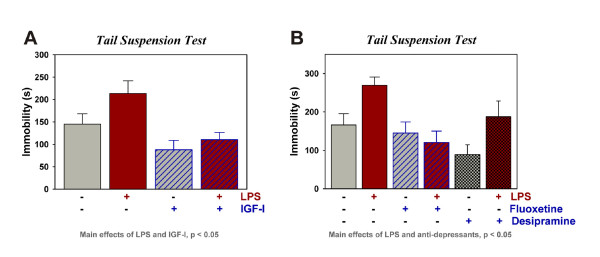
**IGF-I displays anti-depressant activity in the presence or absence of LPS**. IGF-I (1000 ng) or (PBS) was administered i.c.v. 30 min before i.c.v. PBS or LPS (10 ng). Depressive-like behavior was assessed as immobility in the TST to define the effect of IGF-I (A), fluoxetine and desipramine (B) on depressive-like behavior. n = 16 to 19 per treatment for data in (A) and n = 8 for 9 for the data in (B)

The TST was performed 9 h post LPS (Figure 1A) using mice treated i.c.v. with PBS or IGF-I (1,000 ng) 30 min prior to PBS or LPS (10 ng). As expected, LPS-treated mice exhibited an increase in immobility compared to the control groups [F (1,65) = 4.2, p < 0.05]. IGF-I significantly decreased the duration of immobility [F (1,65) = 13.1, p < 0.001]. There was no significant interaction. These data indicate that IGF-I attenuated depressive-like behavior in the absence and presence of LPS. The increased immobility that occurs 9 h after LPS administration was inhibited by fluoxetine, a classic selective serotonin reuptake inhibitor (SSRI) anti-depressant, and desipramine, a classic tricyclic anti-depressant, (Figure 1B, p < 0.05 for the main effect of LPS, p < 0.05 for the main effect of antidepressants) supporting this behavioral change after LPS as a depressive-like response. The anti-depressant effect of desipramine mimicked that of IGF-I (suppressing immobility in the absence or presence of LPS), whereas, fluoxetine did not affect immobility in control mice. Collectively, these data establish that central LPS is capable of inducing depressive-like behavior, similar to the established effect of peripheral LPS. More important, the results show that IGF-I shares with classic anti-depressants, the ability to inhibit depressive-like behavior.

### IGF-I does not prevent LPS-induced decreases in body weight, feed intake or social exploration

**Figure 2:**
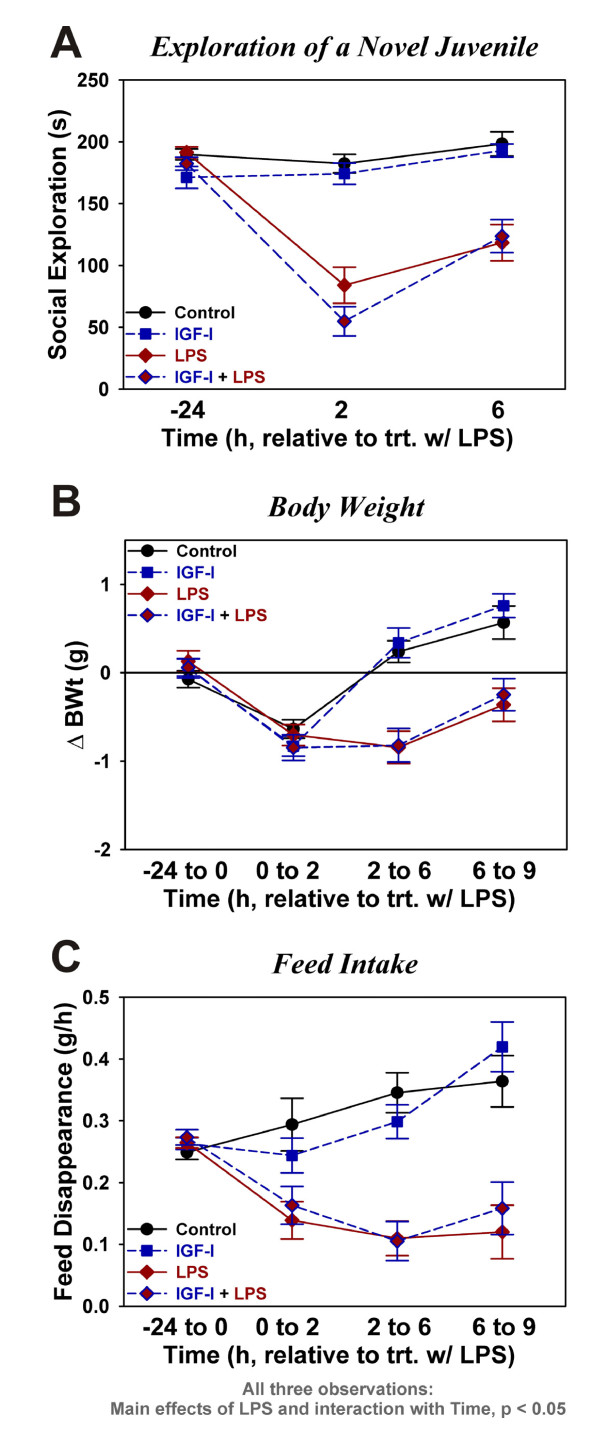
**Central IGF-I does not affect central LPS-induced sickness**. Social exploration, time spent investigating a novel protected juvenile mouse, was assessed as an index of sickness behavior (A). Body weight change (B) was determined over the indicated time intervals relative to LPS treatment. Feed disappearance over the same intervals (C) was used as an estimate of feed intake. Data were analyzed as repeated measures by ANOVA. n = 16 to 19 for the data in (A) and n = 11 to 13 per treatment for data in (B) and (C)

To investigate the effect of i.c.v. administered IGF-I on sickness behavior, social exploration towards a novel juvenile was assessed (Figure 2A). Baseline duration of exploratory activity was similar for all treatment groups, averaging ~3 min over the 5 min test period. LPS significantly decreased exploratory behavior [F (1,130) = 69.5, p < 0.0001] with an LPX × time interaction [F (2,130) = 46.6, p < 0.0001]. At 6 h, LPS-treated mice showed an increase in social activity compared to the 2 h time point, indicating that they were recovering from the sickness. IGF-I did not affect social exploration at either time point. These data show that the effect of IGF-I on duration of immobility in the TST was not mediated by a general decrease in sickness behavior.

IGF-I did not affect body weight or feed intake. All groups of mice lost some weight during the first two hours post-LPS (Figure 2B). LPS-treated mice showed a significant loss of body weight by 6 h, whereas controls showed no additional change in body weight. These data resulted in a significant time [F (3,129) = 25.9, p < 0.0001], LPS [F (1,129) = 41.0, p < 0.0001] and time × LPS interaction [F (3,129) = 14.1, p < 0.0001], but no significant effects for IGF-I. As expected (Figure 2C), LPS-treated mice consumed less feed than controls with significant time [F (3,126) = 7.1, p < 0.001], LPS [F (1,126) = 36.1, p < 0.0001] and time × LPS interaction [F (3,126) = 15.3, p < 0.0001]. IGF-I did not affect feed intake. These data establish that central IGF-I does not alter the sickness response to this dose of LPS.

### IGF-I administration up-regulates BDNF expression in the presence and absence of LPS

**Figure 3:**
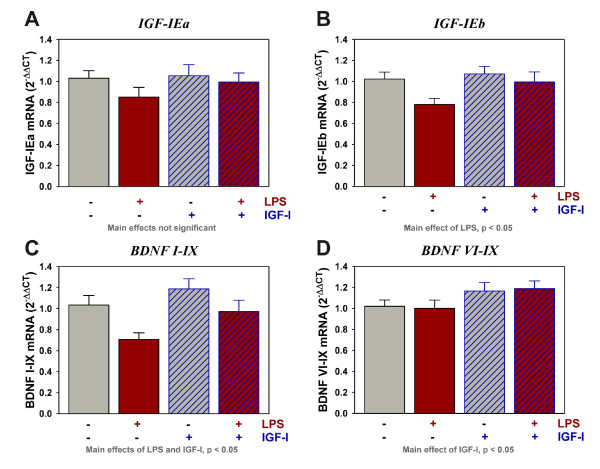
**IGF-I increases BDNF mRNA transcripts in the presence or absence of LPS**. Steady-state mRNA expression for the two 3' classes of IGF-I (A, B) and two distinct BDNF transcripts (C, D) was quantified by real-time rtPCR. Expression was relative to GAPDH, which was used as a housekeeping control gene. n = 10 to 12 per treatment

**Figure 4:**
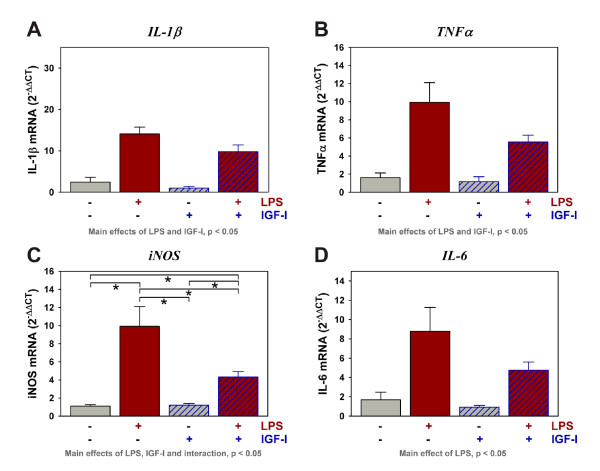
**LPS increases the expression of pro-inflammatory mediators whereas IGF-I attenuates their expression**. Steady-state mRNA expression for IL-1β (A), TNFα (B), iNOS (C) and IL-6 (D) was quantified by real-time rtPCR. Expression was relative to GAPDH. *p < 0.05 following mean separation. n = 10 to 12 per treatment

To assess the possible inducing effect of IGF-I on neuroprotective factors, expression of IGF-I and BDNF were quantified in the brains of mice. The major IGF-I transcript in the mouse brain, IGF-IEa, was unaffected by either LPS or IGF-I (Figure 3A). In contrast, IGF-IEb was reduced by LPS [F (1,45) = 4.5, p < 0.05] (Figure 3B) but similar to IGF-IEa, IGF-IEb was not significantly changed by IGF-I. BDNF is synthesized from multiple transcripts, all of which are expressed in the brain. LPS differentially regulated BDNF transcripts. BDNF I-IX (Figure 3C) was decreased by LPS [F (1,44) = 8.8, p < 0.005], whereas BDNF VI-IX was unaffected by LPS (Figure 4D). Transcripts for both BDNF I-IX [F (1,44) = 5.2, p < 0.05] and BDNF VI-IX [F (1,45) = 5.2, p < 0.05] were elevated by IGF-I. These important results suggest that elevated BDNF expression could be one of the mechanisms underlying IGF-Is' ability to diminish depressive-like behavior of mice.

### IGF-I administration decreases expression of inflammatory proteins

Behavioral changes induced by LPS are known to be mediated by inducing expression of pro-inflammatory mediators. Here we tested the ability of IGF-I to modulate the expression of pro-inflammatory factors induced by LPS. As expected, central LPS (Figure 4A and 4B) significantly increased mRNA expression of IL-1β [F (1,44) = 62.6, p < 0.0001] and TNFα [F (1,45) = 30.4, p < 0.0001]. Central IGF-I attenuated the LPS induction of both IL-1β [F (1,44) = 4.9, p < 0.05] and TNFα [F (1,45) = 4.4, p < 0.05]. LPS also increased iNOS [F (1,45) = 31.0, p < 0.0001], and LPS-dependent iNOS expression was significantly suppressed by central IGF-I [F (1,45) = 6.6, p < 0.05] with a statistically significant interaction between IGF-I and LPS [F (1,45) = 7.1, p < 0.05] (Figure 4C). LPS similarly induced expression of IL-6 [F (1,45) = 12.5, p < 0.05]. Although IGF-I appeared to reduce expression of IL-6, this effect did not reach statistical significance. These data indicate that central IGF-I is able to attenuate glial activation, evidenced by its effect on pro-inflammatory cytokine expression.

**Figure 5:**
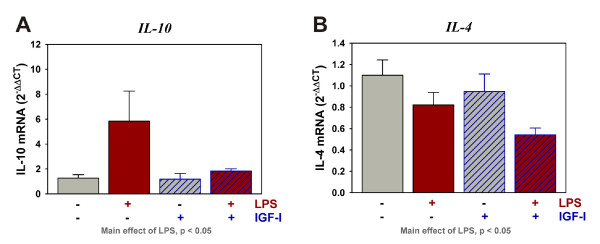
**LPS differentially regulates anti-inflammatory cytokine expression, but IGF-I does not alter the expression of IL-10 or IL-4**. Steady-state mRNA expression for the anti-inflammatory makers IL-10 (A) and IL-4 (B) was quantified by real-time rtPCR. Expression was relative to GAPDH, which was used as a housekeeping control gene. n = 10 to 12 per treatment

To further investigate the mechanism by which IGF-I is acting within the brain, we measured mRNA expression of anti-inflammatory factors (Table 1). Similar to the pro-inflammatory cytokines, IL-10 expression was increased [F (1,45) = 5.1, p < 0.05] whereas IL-4 [F (1,44) = 7.2, p < 0.05] was decreased by LPS. Expression of neither cytokine was significantly altered by IGF-I (Figure 5A and 5B). These data indicate that IGF-I's ability to attenuate glial activation was only observed as an effect on pro-inflammatory cytokine expression.

### IGF-I decreased GFAP but not COX2 expression

**Figure 6:**
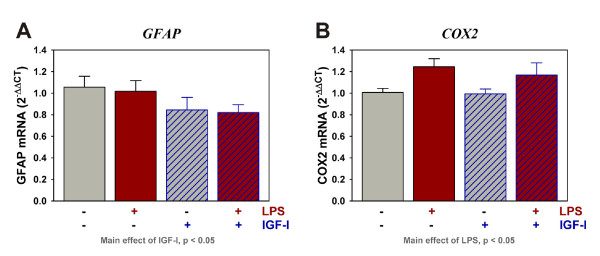
**IGF-I decreases the expression of GFAP but not COX2**. Steady-state mRNA expression for GFAP (A) and COX2 (B) were quantified by real-time rtPCR. Expression was relative to GAPDH, which was used as a housekeeping control gene. n = 10 to 12 per treatment

Glial fibrillary acidic protein (GFAP) expression was quantified to determine if IGF-I influenced astroglial activation. As shown in Figure 6, LPS did not alter expression of GFAP at 9 h. However, central treatment with IGF-I significantly decreased the expression of GFAP [F (1,44) = 4.3, p < 0.05]. As expected, LPS increased COX2 expression [F (1,44) = 7.6, p < 0.01], but this increase was not modulated by IGF-I.

## Discussion

The most important finding of this study was that central administration of 1,000 ng IGF-I significantly reduces the duration of immobility after the injection of either vehicle or LPS. This is the first evidence to establish that IGF-I tempers the innate immune response within the brain following central administration of LPS. IGF-I acts to reduce expression of central inflammatory markers while increasing expression of the neurotrophin BDNF. There was no statistical interaction between IGF-I and LPS on depressive-like behavior (immobility during the TST), thus IGF-I did not act to specifically block LPS-induced depressive-like behavior. However, these data do establish that the anti-depressant activity of IGF-I is present in both naïve and LPS-challenged mice. To assure that immobility in the TST could be used to model depressive behaviors, we confirmed that immobility was responsive to classic anti-depressants, fluoxetine and desipramine. Both of these drugs had anti-depressant activity 9 h after LPS, fluoxetine eliminating the LPS response and desipramine acting more like IGF-I; i.e. lowering immobility in the absence or presence of LPS. These findings are in line with other work with fluoxetine and desipramine [33,34]. The anti-depressant effect of IGF-I was also observed in the absence of LPS, which is consistent with other studies with rodents where increasing IGF-I activity decreases depressive-like behaviors, including diminished immobility during the FST or TST [26-28].

Activation of the innate immune system of the brain contributes to pathophysiologic changes that occur in depression due to the generation of inflammatory mediators including nitric oxide, via iNOS, and pro-inflammatory cytokines such as IL-1β and TNFα. Although tested at much higher doses (500 to 25,000 ng) than used for the current experiment, numerous experiments have shown that LPS given centrally induces steady-state mRNA expression of cytokines that are directly related to an increase in the content of the transcribed proteins n the rodent brain [35-42]. Our results showed that as little as 10 ng LPS induces expression of cytokines and iNOS. In contrast, central administration of IGF-I reduced the expression of IL-1β, TNFα and iNOS (Figure 4). The effects of LPS and IGF-I on pro-inflammatory cytokine and iNOS expression directly paralleled depressive-like behavior. Although having a similar inhibitory trend, central IGF-I did not significantly affect IL-4, IL-6 or IL-10. IGF-I also decreased the expression of GFAP. GFAP expression is recognized as a marker of astrocyte activation [43] and LPS has been shown to increase GFAP expression [44] and cytokine expression [45] by astrocytes. However, our low i.c.v. dose of LPS was not capable inducing GFAP expression. These data do, however, provide strong evidence that the general degree of activation of the innate immune system within the brain was depressed by IGF-I.

Fluoxetine and desipramine, which blocked the increase in duration of immobility caused by LPS, both have anti-inflammatory activity [46-49]. This finding supports a possible direct role of the innate immune system in depressive-like behavior and the possible importance of the anti-inflammatory impact of IGF-I on behavior. Whether the decrease in cytokine expression induced by IGF-I was causative, remains speculative. However, administration of the anti-inflammatory agent, minocycline, similarly depressed cytokine expression in the brain of mice and this anti-depressant was more effective in diminishing depressive-like behavior than decreasing the sickness response [12]. Similar to the results reported here, this finding indicated dissociation between sickness and depressive-like behavior. Such dissociation was also found with cancer patients wherein paroxetine, a SSRI class anti-depressant, had little activity against neurovegatative symptoms of sickness following IFNγ treatment, whereas symptoms of depression were more responsive to paroxetine [8].

The neurotrophic hypothesis of depression considers that functional changes in existing neurons and their synapses (neuronal plasticity) and the organism's ability to adapt/respond to the environment via neurogenesis or reorganization of neuronal networks (brain plasticity) are dysfunctional because of a deficient degree of neurotrophic support and classic anti-depressants, such as fluoxetine, reactivate plasticity in the adult brain [50,51]. The IGF system is one of the most characterized neurotrophic networks yet described with IGF-I inducing the proliferation, survival, differentiation and maturation of all cells of the central nervous system [52]. In addition, IGF-I directly supports neuronal and brain plasticity [53], key components of the neurotrophic hypothesis. In the brains of naïve mice, IGF-I mRNA is expressed primarily by neurons [54-56]. IGF-IEa is the primary transcript in the rodent brain throughout development although IGF-IEb (also sometimes referred to as IGF-IEc) expression can be induced [57]. We confirmed the prominence of IGF-IEa above IGF-IEb in the mouse brain (Table 1). The effect of central administration of LPS on IGF-I expression has not been reported. Although not a major source of IGF-I in the naïve rodent brain, LPS depressed the secretion of IGF-I from cultured microglia [58]. This inhibitory effect of LPS agrees with the reduction in IGF-IEb mRNA expression in the brain reported here.

Pro-inflammatory cytokines induce IGF resistance of multiple cell types (reviewed in [13]), but this possibility has never been tested with cells of the central nervous system. Whether activation of the immune system by central LPS and the subsequent expression of pro-inflammatory cytokines decrease IGF-I activity in the brain is unknown. However, it is important to note that addition of exogenous IGF-I strikingly diminished depressive-like activity. Thus, our IGF-I treatment paradigm may have been successful in LPS-challenged mice because it either restored the effect of lowered IGF-I mRNA expression, caused by LPS, or overcame the effect of cytokines-induced IGF resistance. Further work is necessary to clarify the mode by which LPS/cytokines-induced and IGF-I-reduced depressive-like behavior.

In a previous study, 1,000 ng IGF-I attenuated the decrease in exploration of a novel object induced by i.c.v. administration of 100 ng LPS to CD-1 mice [23]. This was accompanied by an IGF-I induced reduction in the body weight loss that occurred following LPS treatment. IGF-I also attenuated the decreased social exploration induced by centrally administered TNFα or IL-1β although IGF-I was more potent against TNFα than IL-1β and only blocked the reduction in body weight loss that was induced TNFα [24]. These results were replicated in a different set of experiments using only TNFα [25]. Since the decrease in social exploration caused by inflammation is essentially mediated by IL-1β, with TNFα having only an accessory role [59], the present results, wherein IGF-I did not block sickness, are not necessarily in contradiction with our previous results on the effects of IGF-I on sickness behavior. A sub-optimal very low dose of LPS was chosen for the current study in order to elicit a minimal and transient bout of sickness and thus a lower metabolic challenge to the mice. In addition, this very low dose of LPS was carefully chosen not to induce IGF-I sensitive sickness behavior, but to cause reliable depressive-like behavior at a time at which the mice are recovering from sickness. The time interval of 9 h post-LPS was shorter than the 24 h time interval that was used in our previous experiments with LPS administered i.p. [12] for the simple reason that a low i.c.v. dose of LPS caused more transient behavioral alterations than systemically administered LPS. The important finding of this work clearly still remains that IGF-I decreased depressive-like behavior independent of an effect on the sickness response. This indicates that although linked, sickness behaviors and depressive-like behaviors of mice have subtle yet distinct underlying etiologies. This dissociation between sickness behavior and depression has already been noted in both preclinical [12] and clinical studies [8].

The role of BDNF in depression was recently reviewed by several investigators. A genetic link has been identified between BDNF variants and response of humans to anti-depressants [60] and lower BDNF expression was associated with the development of major depressive disorder [61]. Using animal models, anti-depressant administration, therapeutic regimes such as exercise, and IGF-I all increased BDNF expression within the brain. BDNF and BDNF receptor (TrkB) deficient mice or those carrying a human variant, associated with resistance to anti-depressants, did not respond to anti-depressants. BDNF deficiency in the forebrain also caused a deficit in the TST [62,63] and anti-depressants induced BDNF expression [51]. These studies clearly identify a role for BDNF in depressive-like behavior; lower BDNF increased depressive-like behavior whereas higher BDNF lowered depressive-like behavior. Relative to the current work, LPS either decreased or did not change BDNF expression within the hippocampus [64-67] or decreased expression in the whole brain 3 days after i.p. LPS [68]. One of the hottest aspects of BDNF biology is the differential expression and regulation of specific transcript; all of which produce the same mature protein. The studies mentioned above quantified total BDNF expression. However, transcripts initiating from exons I, II and III are expressed predominantly in neurons whereas transcripts initiating from exons IV, V and VI are expressed by both neurons and astrocytes within the naïve brain [69]. Normal expression of all transcripts may be necessary for wellness, as a knockout of even one of the 9 BDNF transcripts caused depressive-like behavior of mice [70]. Duloxetine, an SNRI class anti-depressant, increased the expression of only 4 of the 9 transcripts [71]. Clearly, considerable effort is needed to clarify the regulation of BDNF expression, especially relative to immune activation where little is known except that TNFα induced transcript IV expression in cultured astrocytes, whereas LPS and IL-1β did not change expression [72]. There were no reports of IGF-I regulating the expression of specific BDNF transcripts. Here we show that LPS decreased expression of the exon I, not exon VI, driven BDNF transcript, whereas IGF-I increased expression of both transcripts. These specific transcripts were chosen since exon I was known to be neuron-specific, but exon VI transcripts were also present in astrocytes. It was tempting to speculate that LPS, by some uncharacterized mechanism, decreased neuronal, but not astrocyte, expression of BDNF as part of its ability to induce depressive-like behavior. Proof of this relationship awaits further evidence. IGF-I increased expression of BDNF I and VI; transcripts found in neurons or both neurons and astrocytes, respectively; and IGF-I decreased depressive-like behavior. Elevated BDNF expression may be a major part of the mechanism by which IGF-I acted as an anti-depressant.

Since BDNF has anti-depressant activity, the increase in BDNF expression may be associated with the anti-depressant actions following central administration of IGF-I. However, IGF-I also enhances BDNF signaling within neurons. We had previously shown that IGF-I sensitizes cortical neurons to BDNF: increasing BDNF induction of Erk phosphorylation and synergizing with BDNF to decrease calcium uptake following treatment with glutamate [73]. Those data support earlier work showing that IGF-I and BDNF synergize to support neuron survival [74]. Together they clearly indicate that IGF-I and BDNF work together within the brain. Also, IGF-I directly supports neuronal plasticity [53] as does BDNF (reviewed in [51]). It will be interesting to determine if IGF-I and BDNF synergize to increase neuronal plasticity thus offsetting the well-characterized loss of synaptic plasticity following treatment with LPS [75-78]. Such a finding may further define a precise mechanism by which either growth factor regulates depressive-like activities.

## Conclusions

Data in this report further establish that central administration of IGF-I results in anti-depressant-like activity. This main effect of IGF-I, on immobility during the TST, confirms that the anti-depressant activity of IGF-I is similar across naïve and LPS-challenged mice. Importantly, administration of IGF-I increases the expression of BDNF while decreasing the expression of inflammatory proteins; similarly across naïve and LPS-challenged mice. These data form the basis for future work defining the mechanism for IGF-I's anti-depressant activity. The anti-depressant activity of IGF-I may have clinical implications for psychiatric conditions with or without the presence of inflammatory diseases.

## Abbreviations

**BDNF**: brain-derived neurotrophic factor; **COX**: cyclooxygenase; **CUS**: chronic mild unpredictable stress; **FGL**: fibroblast growth factor loop; **FST**: forced swim test; **GFAP**: glial fibrillary acidic protein; **i.c.v**: intracerebroventricular; **IFN**: interferon; **IGF**: insulin-like growth factor; **IL**: interleukin; **Inos**: inducible nitric oxide synthase; **JNK**: c-Jun N-terminal kinase; **LPS**: lipopolysaccharide; **NCAM**: neural cell adhesion molecule; **PBS**: phosphate buffered saline; **PCR**: polymerase chain reaction; **s.c**.: subcutaneous; **TNF**: tumor necrosis factor; **TST**: tail suspension test

## Competing interests

RD and KWK have received honoraria from Astra-Zeneca, RD has received honoraria from Bristol-Myers Squibb and Lundbeck Laboratories. RD works as a consultant for Lundbeck Laboratories

## Authors' contributions

RHM designed the experiments, performed surgery, treated animals, performed behavior tests, analyzed data and wrote much of the manuscript. SP performed surgery, treated animals, performed behavior tests, analyzed data, did all the PCR analyses and wrote some of the manuscript. KWK and RD helped in the design and interpretation of the experiments and editing the manuscript. All authors have read and approved the final version of this manuscript.
